# Vertical distribution of brittle star larvae in two contrasting coastal embayments: implications for larval transport

**DOI:** 10.1038/s41598-020-68750-4

**Published:** 2020-07-21

**Authors:** Morgane Guillam, Claire Bessin, Aline Blanchet-Aurigny, Philippe Cugier, Amandine Nicolle, Éric Thiébaut, Thierry Comtet

**Affiliations:** 1 Sorbonne Université, CNRS, Station Biologique de Roscoff, Laboratoire Adaptation Et Diversité en Milieu Marin, ADMM, CS90074, 29688 Roscoff Cedex, France; 2 Ifremer, Centre de Bretagne, Département Dynamiques des Ecosystèmes Côtiers (DYNECO), Laboratoire d’Ecologie Benthique Côtière (LEBCO), Technopole Brest Iroise, CS 10070, 29280 Plouzané, France; 3 ENSTA Bretagne, Pôle STIC/OSM, 2 rue François Verny, 29806 Brest Cedex 9, France

**Keywords:** Marine biology, Ecology

## Abstract

The ability of marine invertebrate larvae to control their vertical position shapes their dispersal pattern. In species characterized by large variations in population density, like many echinoderm species, larval dispersal may contribute to outbreak and die-off phenomena. A proliferation of the ophiuroid *Ophiocomina nigra* was observed for several years in western Brittany (France), inducing drastic changes on the benthic communities. We here studied the larval vertical distribution in this species and two co-occurring ophiuroid species, *Ophiothrix fragilis* and *Amphiura filiformis*, in two contrasting hydrodynamic environments: stratified in the bay of Douarnenez and well-mixed in the bay of Brest. Larvae were collected at 3 depths during 25 h within each bay. In the bay of Brest, all larvae were evenly distributed in the water column due to the intense vertical mixing. Conversely, in the bay of Douarnenez, a diel vertical migration was observed for *O.* *nigra*, with a night ascent of young larvae, and ontogenetic differences*.* These different patterns in the two bays mediate the effects of tidal currents on larval fluxes. *O. fragilis* larvae were mainly distributed above the thermocline which may favour larval retention within the bay, while *A.* *filiformis* larvae, mostly concentrated near the bottom, were preferentially exported. This study highlighted the complex interactions between coastal hydrodynamics and specific larval traits, e.g. larval morphology, in the control of larval vertical distribution and larval dispersal.

## Introduction

Echinoderms play key roles in the structure and the functioning of many marine ecosystems, and are known for large variations in population density, alternating periods of outbreaks and periods of die-offs^1^. These rapid and drastic changes in their abundances can have important ecological consequences on the diversity, resilience and functioning of these ecosystems^1^. The causes of such extreme phenomena are still poorly known, but may be of anthropogenic origin, such as the degradation of water quality, the overexploitation of marine resources or even the introduction of invasive species^1,2^. Besides these primary causes, particular life-history traits of echinoderm species (e.g. external fertilization, planktotrophic larval development) may modulate, either amplifying or reducing, the variations of abundances of adult populations^1^. In particular, shifts in larval survival, in response to changes in food supply, or larval dispersal may contribute to population decline or increase, while larval connectivity between populations may promote the recovery of depleted populations or enhance the extent of population increases^3–5^.

In the European coastal waters, such phenomena have been described among populations of Echinoidea [e.g. *Echinocardium cordatum* (Pennant, 1777), *Paracentrotus lividus* (Lamarck, 1816)]^6–8^, Asteroidea [e.g. *Asterias rubens* Linnaeus, 1758, *Luidia sarsi* Düben & Koren in Düben, 1844, *Luidia ciliaris* (Philippi, 1837)]^9^, and Ophiuroidea [e.g. *Amphiura filiformis* (O.F. Müller, 1776)]^10^. For instance, a high proliferation of *Ophiocomina nigra* (Abildgaard in O.F. Müller, 1789), a common native species of the coasts of north-western Europe, has recently been reported in two coastal embayments of Brittany (France), i.e. the bay of Douarnenez and the bay of Brest, over the last twenty years^11,12^. Blanchet-Aurigny et al.^11,13^ argued that changes in food supply to adults in response to eutrophication, with the proliferation of green macroalgae, could be the primary cause of the population outbreak of this species which displays a wide trophic plasticity. Therefore, following a fivefold increase in density and a threefold increase in biomass in the bay of Brest, *O.* *nigra* became the predominant primary consumer within the benthic habitats of this bay with strong impacts on the community structure and the bentho-pelagic coupling. One major impact was the significant decline of the populations of the co-occurring brittle-star *Ophiothrix fragilis* (Abildgaard in O.F. Müller, 1789) which was previously dominant in the two bays^11,12^ (Blanchet-Aurigny, pers. obs. 2016), inducing potential consequences on ecosystem functioning^11,13–15^.

Most species of ophiuroid have a bentho-pelagic life cycle including a planktonic larval stage and two bottom-dwelling juvenile and adult stages^16,17^. In particular, ca. 34% of them have planktotrophic ophiopluteus larvae^1^ which feed on suspended particles^18^, allowing them to spend several days to weeks in the plankton. For such species like *Ophiocomina nigra*, *Ophiothrix fragilis* and *Amphiura filiformis*, with planktonic larval durations ranging between 3 and 6 weeks, larval dispersal is an important determinant of population dynamics which influences the sustainability of local populations and contributes to exchange between neighbouring populations or the expansion to new areas^19^. Dispersal is even more important in the context of population outbreaks and die-offs by being able to amplify or mitigate these changes at different spatial scales, such as the amplification of a local proliferation at a regional scale^1,4,20^, which calls for more investigations of the larval features of such species^21,22^.

Larval dispersal depends on complex interactions between the physical properties of the environment, mainly the local hydrodynamics (advection and diffusion), and different biological traits including spawning behaviour, planktonic larval duration, active larval behaviour, larval mortality rate and settlement behavior^23–26^. As currents vary in direction and speed with depth, different larval behaviours (e.g. regulation of vertical position, active vertical migration, passive sinking) may promote differences in local-scale horizontal distributions^27^. Larval ability to control their vertical distribution depends on larval swimming velocities which are highly variable among species, from cm s^−1^ for decapod crustaceans to mm s^−1^ for bivalves, polychaetes and echinoderms^28^, sinking velocities (when larvae stop swimming), which depend on larval buoyancy, and hydrodynamic parameters such as the intensity of vertical mixing^29,30^. Although vertical migratory behaviour has been well documented in decapod and bivalve larvae, this process is poorly known for echinoderm larvae, and more specifically for ophiuroid larvae.

To better assess the larval features and dispersal abilities of *Ophiocomina nigra* and two common co-occurring ophiuroid species, i.e. *Ophiothrix fragilis* and *Amphiura filiformis*, in the context of the increase of population density of *O.* *nigra* in Brittany, we compared their larval vertical distribution in two contrasting areas of western Brittany, i.e. a well-mixed and highly energetic system, the bay of Brest, and a stratified system, the bay of Douarnenez, both interconnected to the Iroise Sea. The vertical distribution of larvae is described according to the larval developmental stages and the day/night and tidal cycles over two 25-h time-series, and data are used to estimate larval fluxes. The mechanisms involved in the regulation of larval vertical distribution and the role of the variability in near-shore hydrodynamics on larval dispersal are discussed.

## Results

### Light intensities and hydrological structure

**Figure 1:**
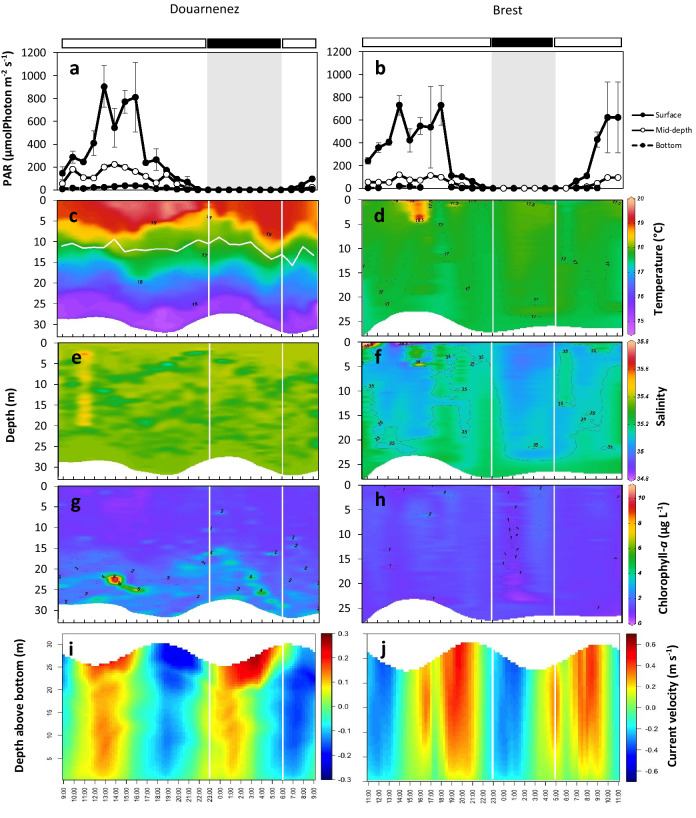
Vertical profiles of environmental parameters in the bay of Douarnenez (left column) and the bay of Brest (right column). (**a**, **b**) Light intensity: each point corresponds to the mean of PAR values at a given hour and layer, and the vertical bars are the standard deviation. Grey shaded area indicates nighttime. (**c**, **d**) Temperature, (**e**, **f**) salinity, (**g**, **h**) chlorophyll-*a* concentration, and (**i**, **j**) current velocity and direction; positive values indicate eastward currents. Horizontal black and white bars indicate nighttime and daytime, respectively. Horizontal white line in c is the thermocline depth. Vertical white lines correspond to times of sunset and sunrise (see “Methods” section).

The surface light intensities followed a similar pattern in both sites, with a peak of intensity from 13:00 to 16:00 in the bay of Douarnenez (max. of 903 µmol Photon m^−2^ s^−1^) and from 14:00 to 18:00 in the bay of Brest (max. of 729 µmol Photon m^−2^ s^−1^), followed by a sharp decrease until to reach values less than 0.15 µmol Photon m^−2^ s^−1^ during the night (Fig. 1a, b). In the bay of Brest, the surface PAR increased rapidly the next morning from 8:00 (Fig. 1b). The light intensities at mid-depth and at the bottom followed the same diel pattern but remained weak; maximum values at mid-depth reached about 220 µmol Photon m^−2^ s^−1^ in the bay of Douarnenez and 120 µmol Photon m^−2^ s^−1^ in the bay of Brest while maximum values in near-bottom waters never exceeded 40 µmol Photon m^−2^ s^−1^ in both bays.

Vertical profiles of water temperature differed between the two bays. In the bay of Douarnenez, the water column was stratified throughout the survey with a thermocline located between 8.9 and 15.6 m depth (Fig. 1c). The water temperature ranged from 14.6 °C at the bottom to 19.8 °C at the surface with maximal value reported during daytime in response to solar radiation. Conversely, in the bay of Brest, no vertical stratification was observed. The temperature varied from 16.6 to 17.5 °C according to the tidal cycle, and peaked to 19.3 °C in the first 5 m of water between 14:00 and 16:00 (Fig. 1d). Salinity remained homogeneous within the water column in the two sampling sites ranging from 34.8 to 35.8 in the bay of Douarnenez and from 34.5 to 35.6 in the bay of Brest (Fig. 1e, f). In the bay of Brest, salinity varied with the tidal cycle with lower values measured during low tide when temperature was higher. Chlorophyll-*a* concentrations varied from 0.6 to 10.8 µg L^−1^ in the bay of Douarnenez and from less than 0.1 to 4.04 µg L^−1^ in the bay of Brest, with the lowest values observed in surface waters in both cases (Fig. 1g, h). In particular, chlorophyll-*a* concentrations in the bay of Douarnenez were higher below the thermocline, with a noticeable high concentration observed from 13:00 to 17:00 in the 20–25 m depth interval, reaching more than 10 µg L^−1^ (Fig. 1g).

Modelled current velocities ranged between 2.10^–5^ and 0.27 m s^−1^ in the bay of Douarnenez and between 2.10^–5^ and 0.48 m s^−1^ in the bay of Brest (Fig. 1i, j). In the bay of Douarnenez, current velocity varied according to depth: it was higher at the surface layer and decreased towards the bottom (Fig. 1i). Current direction changed according to the tide, with westward currents during the ebb and eastward currents during the flow (Fig. 1i). A particular feature was observed during the night, between 3:00 and 7:00: a dynamic stratification was observed with an inversion of current direction at 9 m depth (20–25 m above the bottom) (Fig. 1i). In the bay of Brest, the current velocities were vertically homogeneous, except in the benthic boundary layer (1–2 m above the bottom) with westward currents during the ebb tide and eastward currents during the flow (Fig. 1j).

### Composition of larval communities

**Figure 2:**
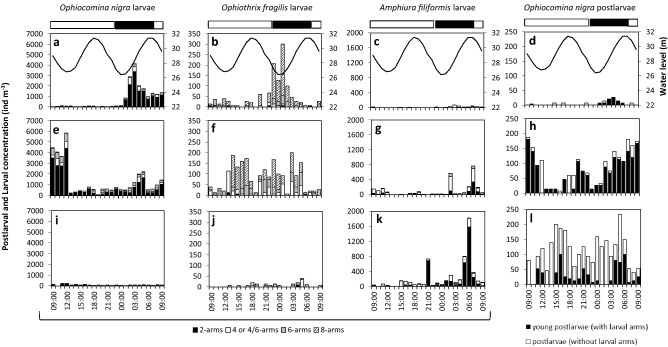
Larval and postlarval concentrations of *Ophiocomina nigra,* and larval concentrations of *Ophiothrix fragilis* and *Amphiura filiformis* at three depths in the bay of Douarnenez in relation to diel and tidal cycles. (**a**–**d**) Surface (3 m), (**e**–**h**) mid-depth (10–12 m), and (**i**–**l**) bottom (24–26 m). The black line represents the water level (m). Horizontal black and white bars indicate nighttime and daytime, respectively. The proportion of the different developmental stages is indicated.

Larvae of five ophiuroid species were observed in both sites. *Ophiocomina nigra, Ophiothrix fragilis*, and *Amphiura filiformis* showed the highest concentrations. The two other species *Ophiura ophiura* (Linnaeus, 1758) and *Ophiactis balli* (W. Thompson, 1840) were much rarer, representing 3% and 0.03%, respectively, of the total larval abundance of the two bays. These two species were not considered further in the present study. In the bay of Douarnenez, larvae of *O. nigra* dominated, representing 82% of the total larval abundance (Fig. 2), with *O. fragilis* and *A. filiformis* representing 5% and 13% of the larval pool, respectively. *O. nigra* was mainly represented by 2-arm larvae which accounted for more than 72% of its larvae, when 8-arm larvae accounted for only 1%. *A. filiformis* had a similar population structure with a dominance of 2-arm larvae (53% of the total larval abundance, against 6% for 6-arm larvae). Conversely, the population of *O. fragilis* larvae was mainly composed of 8-arm larvae (61%), with only 2% of 2-arm larvae. Postlarvae of *O. nigra* were also observed in the bay of Douarnenez, half of them still bearing larval arms (Fig. 2d, h, l).

**Figure 3:**
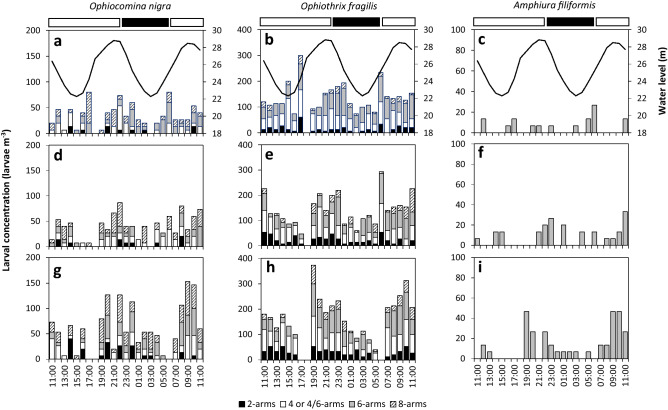
Larval concentrations of *Ophiocomina nigra*, *Ophiothrix fragilis* and *Amphiura filiformis* at three depths in the bay of Brest in relation to diel and tidal cycles. (**a**–**c**) Surface (1.5 m), (**d**–**f**) mid-depth (10 m) and (**g**–**i**) bottom (17–26 m). The black line represents the water level (m). Horizontal black and white bars indicate nighttime and daytime, respectively. The proportion of the different developmental stages is indicated.

In the bay of Brest, larvae of *O. fragilis* dominated the larval pool with 78% of total larval abundance, most of them being 4/6-arm larvae (38% of *O.* *fragilis* larval pool) and 6-arm larvae (36% of *O.* *fragilis* larval pool) (Fig. 3). *O. nigra* and *A. filiformis* represented only 23% and 4% of ophiuroid larvae, respectively. Most of them were later stages (6–8 arms for *O.* *nigra* and 6 arms for *A. filiformis*) (Fig. 3).

### Tidal variations in larval concentrations

In both bays, the depth-cumulated larval concentrations for the three species varied according to the tidal cycle (See Supplementary Fig. S1 online). In the bay of Douarnenez (Fig. 2a–c, e–g, i–k), maximal larval concentrations occurred around the low tide for *O. nigra* and *O. fragilis* while they were observed around high tide for *A. filiformis*. In the bay of Brest, maximal larval concentrations occurred around the high tide for all the studied species (Fig. 3).

### Vertical distribution of ophiuroid larvae

**Figure 4:**
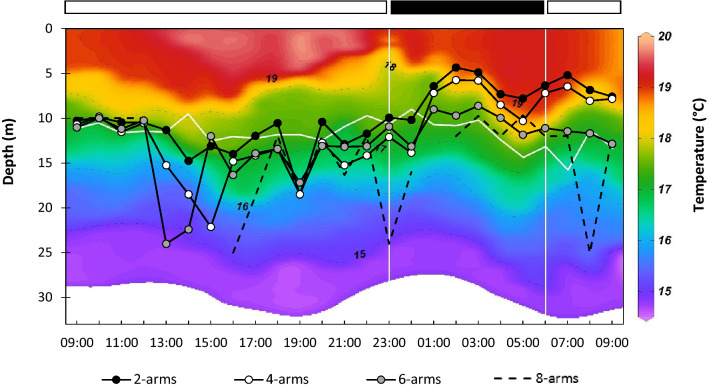
Vertical distribution of the larvae of *Ophiocomina nigra* in the bay of Douarnenez. Mean depth distribution (MDD) of the different larval stages, superimposed on temperature profiles. The horizontal white line corresponds to the location of the thermocline. The horizontal black and white bars indicate nighttime and daytime, respectively.

**Figure 5:**
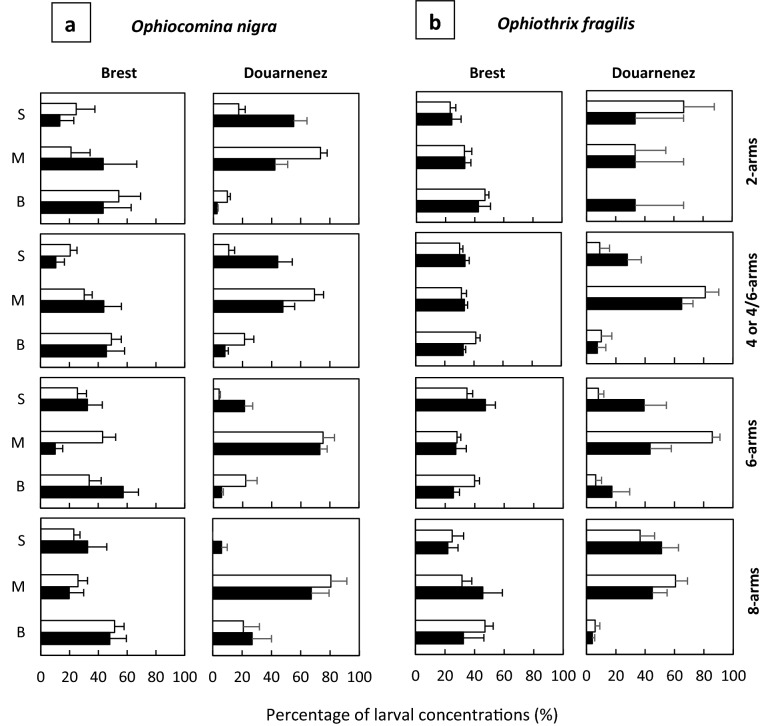
Mean percentage of larval concentration at the three depths (S, surface; M, mid-depth; B, bottom) for *Ophiocomina nigra* (**a**) and *Ophiothrix fragilis* (**b**), during daytime (white bars) and nighttime (black bars), in the bay of Brest and the bay of Douarnenez. Fractions of total counts were computed separately for each larval stage, and averaged for each depth interval across daytime and nighttime samples. Error bars are standard errors.

In the bay of Douarnenez, most larvae of *O. nigra* were observed at the surface (3 m) or at mid-depth (10–12 m) with differences between day and night. Higher larval concentrations were observed at the surface during the night, from 01:00 to the next morning (Fig. 2a, e, i). During daytime, larvae of *O. nigra* were mostly located at or below the thermocline. An increase in the mean depth distribution (MDD) up to 20–25 m depth was observed for 4-, 6-, and 8-arm larvae, a depth where the concentration of chlorophyll-*a* was the highest (Figs. 1, 4). During nighttime, the MDD decreased and larvae were located at lower depths, above the thermocline, especially the 2- and 4-arm larvae (Fig. 4). The mean percentages of larval concentrations of 2, 4-, and 6-arm larvae of *O.* *nigra* in the bay of Douarnenez were significantly higher at the surface at nighttime as compared to daytime (Fig. 5a, Table 1a). No such pattern was observed for 8-arm larvae. Furthermore, differences in MDD were also observed between the different larval stages of *O. nigra* (Fig. 4). These differences were more important at night, but only the difference between 2 and 8-arm stages at night was significant (Table 2) when 2-arm larvae were distributed in the surface layer and 8-arm larvae tended to move below the thermocline. During the day, the differences in vertical distribution between stages were not significant (Table 2). In addition to larvae, postlarvae of *O.* *nigra* were also encountered mostly at depths of 10–25 m (Fig. 2d, h, l). Higher proportions of early postlarvae (i.e. still bearing larval arms) were found at mid-depth, and higher proportions of late postlarvae (i.e. without larval arms) were observed at the bottom layer.

**Table 1:** Comparisons of diel vertical distributions for the different developmental stages of *Ophiocomina nigra*, *Ophiothrix fragilis* and *Amphiura filiformis* in the bay of Douarnenez (a) and the bay of Brest (b).

Comparison	B	*p*	Significant values (/10)
(a)			
*Ophiocomina nigra*			
**2-arms day versus 2-arms night**	**3.9242–10.2370**	**0.0060–0.1406**	**9**
**4-arms day versus 4-arms night**	**3.0721–10.4496**	**0.0054–0.2152**	**9**
**6-arms day versus 6-arms night**	**4.5398–10.2895**	**0.0058–0.1033**	**9**
8-arms day versus 8-arms night	0.6094–7.9716	0.0186–0.6094	1
*Ophiothrix fragilis*			
2-arms day versus 2-arms night	6.06e−10–4.1805	0.1237–1.0000	0
4/6-arms day versus 4/6-arms night	0.5980–7.4278	0.0244–0.7416	1
6-arms day versus 6-arms night	0.6908–6.3985	0.0408–0.7079	2
8-arms day versus 8-arms night	0.6608–5.9050	0.0522–0.7186	0
*Amphiura filiformis*			
2-arms day versus 2-arms night	0.2381–9.1504	0.0103–0.8878	1
4-arms day versus 4-arms night	0.1803–6.6801	0.0354–0.9138	1
6-arms day versus 6-arms night	0.1385–4.8264	0.0895–0.9331	0
(b)			
*Ophiocomina nigra*			
2-arms day versus 2-arms night	0.1127–4.2617	0.1187–0.9452	0
4-arms day versus 4-arms night	0.9854–7.6133	0.0222–0.611	2
6-arms day versus 6-arms night	1.0957–8.4053	0.0150–0.5782	2
8-arms day versus 8-arms night	0.2578–3.9782	0.1368–0.8790	0
*Ophiothrix fragilis*			
2-arms day versus 2-arms night	4.70e−08–1.2523	0.5346–1	0
4/6-arms day versus 4/6-arms night	1.0967–9.8178	0.0074–0.5779	3
6-arms day versus 6-arms night	0.0077–7.4807	0.0237–0.9962	1
8-arms day versus 8-arms night	1.01e−09–2.4995	0.2866–1	0
*Amphiura filiformis*			
6-arms day versus 6-arms night	0.0521–7.1605	0.0279–0.9743	1

The range of the test statistic B, its corresponding *p *values (*p*), and the number of significant *p *values (< 0.05) among the 10 combinations (see “Methods” section) are reported. The bold numbers indicate significant differences.

**Table 2:** Comparisons of the vertical distribution of the different developmental stages of *Ophiocomina nigra*, *Ophiothrix fragilis* and *Amphiura filiformis* in the bay of Douarnenez.

Comparison	Day			Night		
	B	*p*	Significant values (/10)	B	*p*	Significant values (/10)
*Ophiocomina nigra*						
2-arms versus 4-arms	0.7581–4.1939	0.1228–0.6845	0	1.2453–4.5869	0.1009–0.5365	0
2-arms versus 6-arms	0.7326–5.3125	0.0702–0.6933	0	4.9815–8.3919	0.0151–0.0828	0
2-arms versus 8-arms	0.3855–7.3263	0.0257–0.8247	0	**8.8555–12.0262**	**0.0024–0.0119**	**9**
4-arms versus 6-arms	0.0386–2.7937	0.2474–0.9809	0	0.8973–3.8738	0.1441–0.6385	0
4-arms versus 8-arms	0.0640–4.9651	0.0835–0.9685	0	4.4786–10.6771	0.0048–0.1065	2
6-arms versus 8-arms	0.4258–4.8535	0.0883–0.8082	0	2.6250–9.7173	0.0078–0.2691	1
*Ophiothrix fragilis*						
4/6-arms versus 6-arms	0.2352–4.3955	0.1111–0.8891	0	1.3854–4.4813	0.1064–0.5002	0
4/6-arms versus 8-arms	1.8961–5.7955	0.0551–0.3875	0	3.8105–8.5510	0.0139–0.1488	0
6-arms versus 8-arms	0.0805–5.0043	0.0819–0.9606	0	0.8471–3.1427	0.2078–0.6547	0
*Amphiura filiformis*						
2-arms versus 4-arms	0.0517–4.0933	0.1292–0.9745	0	4.5020–9.1455	0.0103–0.1053	0
2-arms versus 6-arms	3.2263–6.0047	0.0497–0.1993	0	4.6089–8.5705	0.0138–0.0998	0
4-arms versus 6-arms	0.4486–4.1189	0.1275–0.7991	0	0.2305–3.2846	0.1935–0.8911	0

Daytime and nighttime were separated to take into account potential diel vertical migration of larvae. The range of the test statistic B, its corresponding *p* values (*p*) and the number of significant *p* values (< 0.01) among the 10 combinations (see “Methods” section) are reported. The bold numbers indicate significant differences.

Most larvae of *O. fragilis* in the bay of Douarnenez were also sampled in surface waters and at mid-depths (Fig. 2b, f, j). However, the mean depth distribution (MDD) of *O. fragilis* larvae varied markedly over time, for all developmental stages (See Supplementary Fig. S2a online) without significant difference in their vertical distribution between day and night (Fig. 5b, Table 1a) and between the developmental stages (Table 2).

In the bay of Douarnenez, unlike *O. nigra* and *O. fragilis*, the larvae of *Amphiura filiformis* were mostly found at mid-depth and bottom (Figs. 2c, g, k). The MDD was deeper for all developmental stages of this species (See Supplementary Fig. S2b online) and no significant difference in vertical distribution was found between daytime and nighttime (Table 1a) and between developmental stages (Table 2).

In the bay of Brest, the three species had homogeneous larval vertical distributions, with overall similar concentrations at the three depths (Fig. 3). No significant differences were found in the vertical distributions between nighttime and daytime regardless of the species and the developmental stage (Fig. 5a, b, Table 1b).

### Larval fluxes

**Figure 6:**
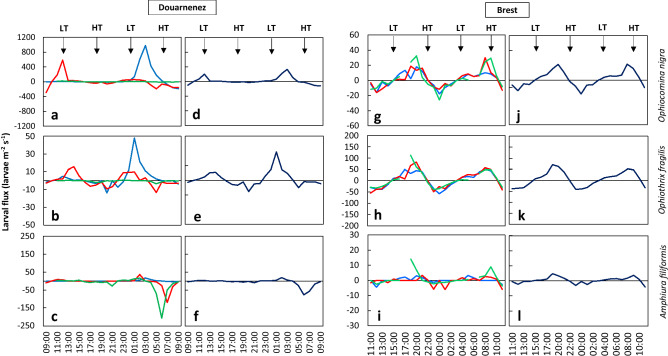
Larval fluxes of *Ophiocomina nigra* (**a**, **d**, **g**, **j**), *Ophiothrix fragilis* (**b**, **e**, **h**, **k**), and *Amphiura filiformis* (**c**, **f**, **i**, **l**), in the bay of Douarnenez (**a**–**f**) and the bay of Brest (**g**–**l**) with the associated water level. Left column (**a**–**c**, **g**–**i**): larval flux at three depths (blue, surface; red, middle; green, bottom). Right column (**d**–**f**, **j**–**l**): mean larval flux throughout the water column. A positive flux corresponds to an eastward flux of larvae into the bay. LT corresponds to low tide and HT corresponds to high tide.

In the bay of Douarnenez, larval fluxes varied with depth: *O. nigra* and *O. fragilis* were characterized by two eastward fluxes of larvae at the surface and mid-depth with an almost null flux near the bottom, with lower values for *O. fragilis* (Fig. 6a, b). *A. filiformis* showed two westward larval fluxes at mid-depth and near the bottom at 6:00 and 7:00, respectively, with very weak fluxes at the surface (Fig. 6c). In the bay of Brest, the larval fluxes were strongly linked to the tidal cycle, with westward larval fluxes during ebb and eastward fluxes during flood, without differences between depths (Fig. 6g–i).

In the bay of Douarnenez, daily larval fluxes of *O. nigra* and *O. fragilis* were positive with 554 and 25 larvae m^−2^ d^−1^ respectively (Fig. 6d–e), and negative for *A. filiformis* (F = − 156 larvae m^−2^ d^−1^; Fig. 6f). In the bay of Brest, the daily larval flux of each species was positive with a daily input of 40, 132 and 7 larvae m^−2^ d^−1^ for *O. nigra*, *O. fragilis* and *A. filiformis*, respectively (Fig. 6j–l).

## Discussion

In the presence of a stratified water column, as in the bay of Douarnenez, the vertical distribution of ophiuroid larvae was not homogeneous. *Ophiocomina nigra* larvae were characterized by an active Diel Vertical Migration (DVM), with young larvae rising towards the surface during the night, and by an ontogenetic vertical migration (OVM)^31^, which indicated that larvae could be able to actively control their vertical position. Conversely, larvae of *Ophiothrix fragilis* and *Amphiura filiformis* were mainly distributed above, or under the thermocline, respectively. The vertical structure of the water column (thermocline, halocline, pycnocline) is known to influence the vertical distribution of zooplankton, including invertebrate larvae. In particular, thermoclines may act as a barrier to vertical migration, although this would differ between species and developmental stages^32–34^. In the bay of Brest, the strong currents and the resulting intense vertical mixing of the water column likely explain the homogeneous vertical distribution of the larvae of the three species, as previously reported for *O. fragilis* larvae in the Dover Strait^35^. Such hydrodynamic conditions may not allow the establishment of active movements^36,37^. Horizontal flow^38^, turbulence^37,39,40^, horizontal and vertical shears^41^, may affect the ability of larvae of various taxa, including echinoderms, to regulate their vertical position by altering their vertical swimming behaviour above certain thresholds.

Different causes may explain the presence of vertical migration of invertebrate larvae. Many larvae exhibit diel vertical migration resulting from negative phototaxis^42,43^, for example to avoid predation by visual predators such as fish^44^, or to protect themselves from ultraviolet exposure^45^ known to alter early development in sea urchins^46,47^. To our knowledge, diel vertical migration in ophiuroid larvae in response to the presence of a predator has not been observed yet, but such behaviour has been evidenced in sea urchins larvae^48^. The vertical distribution of food resources may also influence the vertical distribution of planktotrophic larvae including echinoderms^49–51^. In the bay of Douarnenez, previous observations^52,53^ and our data indicated that the maximum chlorophyll-*a* concentration in July is at the depth of or below the pycnocline (Fig. 1g) which could be a causal factor in the downward migration of *O. nigra* larvae during daytime.

Whatever the cue that triggers the larval behaviour, the regulation of the vertical position of the larvae is controlled by their swimming capacities. The morphology of the larvae, including the number, the length and the type of the larval arms, their elevation angle, the presence of ciliated band on arms and on other parts of the larval body, plays a key role on their swimming abilities, with consequences on their buoyancy, orientation, and vertical velocity^36,54^. Even small morphological changes can result in important changes in swimming performances and vertical positioning^54^. The larval arm elevation angle has the most complex effect on larval vertical velocity^54^ and may explain the difference in vertical behaviour between *O. fragilis* and *O. nigra* in the bay of Douarnenez. The larvae of *O. fragilis* are characterized by two long posterolateral arms with a low arm elevation angle, which provide the larvae with a high weight-carrying capacity and a high swimming speed, but reduce the larval stability in the water column^36^, leading to lesser abilities to control their vertical position, especially in vertical shears. The morphology of *O. nigra* larvae, having multiple larval arms with high arm elevation angle, allows the larvae to have a higher stability in shear conditions, then ensuring larvae of *O.* *nigra* a higher ability to maintain directed movements, in particular an upward swimming, so a better ability to control their vertical position^36,55^. Despite a similar morphology, the larvae of *A. filiformis* have a different vertical distribution to those of *O.* *nigra*, except at their latest developmental stage. This suggests that morphology alone cannot explain their observed distribution in the bottom layers of the water column. Chan et al.^56^ observed a decrease in swimming speed, notably vertical velocity, with increasing age in larvae of *A. filiformis*, which might explain the bottom distribution of old larvae. However, this does not explain why 2-arm larvae of *A.* *filiformis* are also confined in the bottom layer.

If larval morphology differs between species, it also changes during the ophiopluteus development, which may explain in part the ontogenetic vertical migration^55^. In particular, morphological development involves changes in the arm elevation angles of the ophiopluteus larvae, with earlier larvae having higher arm elevation angles and higher ability to maintain vertical movement^55^. Morphological changes in *O. nigra* larvae, such as the lengthening, the development of the arms, or the appearance of a juvenile form in older stages also induce an increase in the larval body density^36^. Conversely, it has been shown in echinopluteus larvae that lipids are accumulated during later developmental stages, reducing their body density^45,57^. The existence of similar processes in ophiopluteus larvae are not known, but the later larval stages of *O. nigra* are known to develop a swimming ciliated band, called epaulettes, on the posterior part of the ophiopluteus allowing an active swimming^18,58^. Changes in stability through ontogeny may thus modulate the response of swimming larvae to turbulence and in turn their ability to control their vertical position as documented in larvae of the sea urchin *Arbacia punctulata*^40^.

Swimming speeds reported in the literature for ophiuroid larvae are commonly weak, lower than 1 cm min^−1^ ^28,59^, in particular for larvae with a high elevation angle, and do not seem consistent with the average distance travelled by the early larvae of *O. nigra*. The existence of vertical flows, although not investigated in our study, could provide an explanation. Clay and Grünbaum (2010)^54^ showed that, in the presence of horizontal shear of vertical flows (i.e. horizontal gradients in vertical flow direction and/or velocity), pluteus larvae of the sand dollar *Dendraster excentricus* (Eschscholtz, 1831), while swimming upward, may be tilted in response to the vertical shear and consequently swim toward the upwelling water. Once taken in the upwelling flow the larval upward velocities may then exceed those of larvae swimming upward in still water. Our observations of the presence of postlarvae of *O. nigra*, without ciliated band, and so without swimming capacity, at the different depths of the water column, also suggested the existence of vertical mixing allowing upward passive transport.

It is clearly established that vertical migration behaviours influence the dispersal pattern of larvae, particularly in bays and estuaries due to vertical differences in the direction and speed of currents^25,60,61^. In the bay of Douarnenez, the vertical position of larvae had an influence on the average larval fluxes. For *O. nigra*, the nocturnal upward migration of many larvae combined with eastward surface currents because of the dynamic stratification induced inward fluxes during the night, which resulted in inward daily fluxes. This suggested that, under these conditions, dispersal of *O. nigra* larvae might be limited. Besides, without any vertical migration behaviour, *O. fragilis* larvae showed also inward daily fluxes, being located above the thermocline or at mid-depth. Conversely, for *A. filiformis*, the bottom position of larvae may lead to their export outside the bay because of the presence of a dynamic stratification from 3:00 to 7:00 when the bottom currents were directed westward, i.e. in offshore direction.

In the bay of Brest, because of the hydrodynamic context, which resulted in the homogeneous vertical distribution of the larvae of the three species, the larvae are passively transported according to the tidal oscillation. In these conditions, according to an asymmetry in flood and ebb currents, this resulted to eastward daily larval fluxes which promote the retention of larvae.

Past studies have shown the link between larval dispersal/local retention and the proliferation of a species^62–64^. The above results showed that under the conditions studied here, the larvae of *O. nigra* may be retained in the two studied bays, whether they show a particular vertical distribution or not. In the particular case of the stratified bay of Douarnenez, the diel vertical migration pattern observed may enhance the larval retention. This may contribute to amplify the local proliferation of *O. nigra* observed for several years in this area. To better assess the role of larvae in the proliferation of *O. nigra* would however require investigating larval dispersal under a wider range of hydrodynamic conditions, for example by using a bio-physical model of larval transport that could include the vertical migration behaviour we observed. Future investigations of the movement features (swimming speed, sinking speed and body density) of ophiuroid larvae will also be needed for a better understanding of the vertical distribution patterns we observed. For all that, larval vertical behaviour, if any, and retention are of course not sufficient to explain the proliferation but more likely may modulate shifts due to other primary causes. For example, *O. fragilis* larvae also appear to be retained and yet populations of this species are declining in the study area. Other factors may contribute to the outbreak of *O. nigra*, such as a better survival rate of planktonic and benthic stages, or a better reproductive effort, that would deserve future investigations.

These last years, significant environmental changes (e.g. water acidification, food concentration)^65,66^ are observed in the bay of Douarnenez and the bay of Brest with potential impacts on the benthic ecosystems. In this context, it would be interesting to understand the current and future consequences of such phenomena on the larval development and ability of larvae to control their vertical position, and therefore, on the population dynamics of ophiuroid species, and by extension, on these benthic communities.

## Methods

### Study area

**Figure 7:**
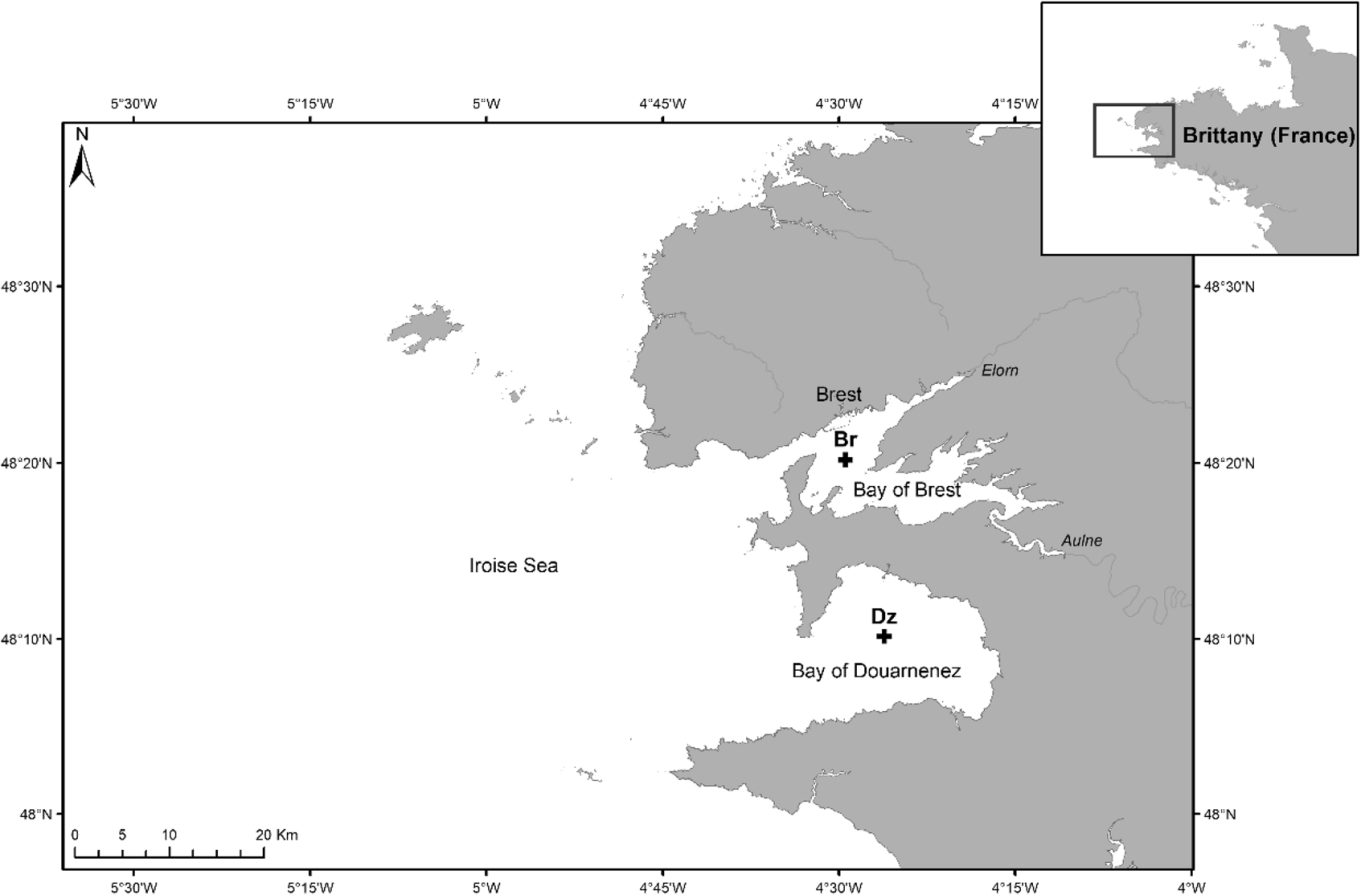
Location of the sampling sites in the bay of Brest (Br) and the bay of Douarnenez (Dz), Brittany (France). The extent of the map represents that of the local high resolution model (Arcgis 10.3.1.).

The bay of Douarnenez, located at the western end of Brittany, is a 350-km^2^ semi-enclosed coastal embayment largely open on the Iroise Sea with a maximum depth of 40 m (Fig. 7). It receives very low freshwater inputs from several streams. In response to weak semi-diurnal tidal currents with a velocity ranging between 0.1 and 1 m s^−1^, a shallow thermal stratification located at about 10–15 m occurs during summer, from June to September, in its central area^53,67^. During this period of time, a maximum of chlorophyll-*a* concentration is found at the depth of or below the thermocline^52,53^. Concomitant with stratification, a coastal front appears at the entrance of the bay and separates the stratified waters of the bay and the well-mixed and colder offshore waters. The bay hosts diverse benthic communities with dense populations of echinoderms, particularly ophiuroids, distributed in various habitats^68^. The highest densities of *O. fragilis* are found in coarse sediments at the opening of the bay and those of *O. nigra* are found on a bottom of maërl and blocks in the north-eastern part of the bay^12^. *A.* *filiformis* inhabits mainly the heterogeneous muddy sand and gravel located in the central part of the bay between 10 and 30 m with densities exceeding 500 ind. m^−2^ ^69^.

The bay of Brest is a 180-km^2^ shallow semi-enclosed bay with an average depth of 8–10 m, connected to the Iroise Sea by a narrow (2 km wide) and deep (40 m) channel. This, together with a high tidal amplitude of 7.5 m in spring tides, induces strong tidal currents reaching 2.6 m s^−1^ and the vertical homogenization of the water column throughout the year^70^. The bay is influenced by the freshwater inputs from two main rivers, the Elorn and the Aulne, which show large seasonal and short-term fluctuations. The bay is characterized by a high diversity in sedimentary features allowing the coexistence of various benthic habitats where ophiuroid species were found in abundance. *O.* *fragilis* is mainly distributed in the southern part of the bay, and *O. nigra* occurs throughout the bay^11^. *A. filiformis* is located near the centre and in the entrance of the bay on fine and muddy sand (observations made in 2013; A. Blanchet-Aurigny and A. Carlier, pers. comm.).

### Sampling

Sampling was conducted onboard the R/V Neomysis during two 25-h cruises on 16–17 July 2014 and 16–17 July 2015 during spring tides in the main central part of the bay of Brest (Br: 48°20′10,26′’N—4°29′27,78′’W; Fig. 7), and the bay of Douarnenez (Dz: 48°10′8,19′’N, 4°26′9,03′’W; Fig. 7), respectively, close to a patch of high abundances of adult ophiuroids^11,12^. Sampling dates lay in the protracted reproductive periods of the target species^71,72^ (Blanchet-Aurigny, unpublished data). Zooplankton samples were collected every hour at 3 different depths (i.e. 1.5–3 m, 10–12 m and 17–26 m corresponding to surface, thermocline or mid-depth, and near-bottom waters, respectively), using a submersible plankton pump (KC Denmark A/S, model 23.570) with a water inflow of 300 L min^−1^. The pump was immersed during 5 min to filter approximately 1.5 m^3^ of water through an 80-µm mesh net. This mesh size allowed to collect all armed larval stages of all 3 target species, which sizes range from 80 µm to 3 mm^58,73–75^. Technical problems prevented us from sampling the bottom layer at 17:00, 18:00, and 06:00 at Br, and 10:00 at Dz. These hours were excluded from the analyses. Samples were preserved on board using 96% ethanol. Real-time vertical profiles of temperature, salinity, PAR (photosynthetically active radiation, 400–700 nm) and chlorophyll-*a* (fluorescence) were performed before each sampling with a Conductivity-Temperature-Depth recorder (CTD Seabird SBE 19) coupled with a Photosynthetically Active Radiation (PAR) sensor (LiCor Ambient Light) and a fluorometer (Seapoint Chlorophyll Fluorometer). Vertical environmental profiles were represented using Ocean Data View software (ODV 4.7.10)^76^.

### Current data

Vertical profiles of instantaneous current velocity and direction were calculated from the 3D hydrodynamic model MARS (Model for Applications at Regional Scale), a 3D primitive equation-free surface model applying the Boussinesq approximation and hydrostaticity^77,78^. Spatial discretization was achieved using the “Arakawa C” differencing scheme in the horizontal, and sigma coordinates in the vertical. The turbulent closure scheme used to compute the vertical turbulent diffusion coefficient was the k–ε model. Horizontal viscosity depends on local mesh dimensions and velocity gradients (see below). It was set to 0.1 m^2^ s^−1^ in the local model (see below), and was computed according to Smagorinsky (1963)^79^ in larger models. Different configurations of the model are available to simulate hydrodynamics and dispersal at different spatial scales, from regional scales (e.g. Bay of Biscay and English Channel)^80^ to local scales (e.g. Dover Strait)^78^. These configurations have been validated from survey data and satellite observations of currents (e.g. ADCP, tidal gauge, VHF radar) and distribution of hydrological parameters (i.e. temperature, salinity)^78,80^.

To properly describe complex hydrodynamics in coastal and nearshore environments, a very high resolution “local” model was used. The model domain extends from 47°57′N to 48°39′N in latitude and from 5°36′W to 4°00′W in longitude. It covers the bay of Douarnenez, the bay of Brest and the Iroise Sea with a horizontal resolution of 170 m and 30 evenly distributed sigma levels. This local model is nested in a larger model of West Brittany with a horizontal resolution of 500 m which provides hydrological open boundaries conditions (i.e. temperature and salinity) and is itself embedded in a regional model covering the Bay of Biscay and the English Channel^81^. For the local model, meteorological conditions (i.e. surface wind stress, atmospheric pressure, air temperature, nebulosity and relative humidity) used to compute momentum and heat exchanges were obtained from the meteorological ARPEGE model at high resolution (spatial resolution of 0.1°, i.e. about 10 km) from the French Meteorological Office Météo-France. Harmonic components of tide at the sea boundaries and bathymetry have been provided by the Service Hydrographique et Océanographique de la Marine (SHOM: The French Navy Oceanographic department). Daily freshwater discharges from the Aulne and Elorn rivers which drive salinity gradients were given by the French water office database. The exit time step of model results was 15 min. The duration of the spin-up period was set to two weeks.

In addition, in the Bay of Brest, vertical profiles of current velocities were measured by an Acoustic Doppler Current Profiler (ADCP; 600 kHz WorkHorse Sentinel) mounted at a surface floating platform during the 16–17 July, 2014. Current velocities were recorded every 2 s throughout the water column in 0.5 m depth bins. These data were used to assess the performance of the “local” high resolution MARS 3D model and its ability to properly characterize the local hydrodynamics and estimate larval fluxes (see Supplementary Methods online).

### Laboratory analyses

All ophiuroid larvae and postlarvae were sorted, identified to species, assigned to a developmental stage and counted following the Frontier’s method (1969)^82^. Briefly, the initial sample was filtered, rinsed and diluted in 150 mL 96% ethanol and three 5-mL replicates were sorted in a Dollfus counting cuvette so that 10% of each sample were observed. Observations were conducted using a dissecting microscope under both transmitted and polarized light to view calcified structures composing the ophiuroid larval skeleton. Larval species identification was based on morphological descriptions given by MacBride (1907)^83^, Chadwick (1914)^58^, Mortensen (1900, 1921, and 1927)^16,17,84^, Narasimhamurti (1933)^73^, and Geiger (1964)^85^, but also on our own reference material (composed of laboratory-reared larvae of *O. nigra*, authors’ unpublished data). The postlarvae have been identified following the description provided by Stöhr (2005)^86^. All ophuiroid larvae were identified and counted, but only the larval concentrations of the 3 dominant species, *Ophiocomina nigra, Ophiothrix fragilis* and *Amphiura filiformis*, were further analysed for their vertical distribution (see below), the abundance of the other species being very low (see Results). Developmental stages were mainly defined based on the number of arms (2, 4, 6 or 8 arms), except for *O. fragilis* where the 2nd and the 3rd pairs of arms develop simultaneously. In this case, the early 6-arm stage is merged with the 4-arm stage in a single 4/6-arm stage. The fully-developed 6-arm stage is distinct and enters in the 6-arm category. The larval development of *A. filiformis* is completed after the 6-arm stage, so no 8-arm stage occurs in this species.

### Data analysis

The depth of the thermocline in the bay of Douarnenez was calculated using a two-layer model of the water column following Planque et al. (2006)^87^. This model assumed that a water column of depth z_b_ is composed of two homogeneous layers, a surface layer of width z_t_ and a bottom layer of width z_b_–z_t_. The thermocline depth z_t_ is described by the following formula^87^:

$$ {\text{z}}_{{\text{t}}} = \frac{{{\text{z}}_{{\text{b}}} \left| {{\text{T}}_{{\text{m}}} - {\text{ T}}_{{\text{b}}} } \right|}}{{\left| {{\text{T}}_{{\text{s}}} - {\text{ T}}_{{\text{b}}} } \right|}} $$

where z_b_ is the water column height, T_m_ is the mean value of temperature from surface to bottom, T_b_ the value of temperature in the bottom layer and T_s_ the value of temperature in the surface layer.

The mean vertical distribution of larvae was assessed by calculating the mean depth distribution (MDD) of each species, each developmental stage and each sampling hour following Tapia et al. (2010)^88^:

$$ {\text{MDD}}_{{\text{j}}} { = }\frac{{1}}{{{\text{N}}_{{\text{j}}} }}\mathop \sum \limits_{{\text{i } = \text{ 1}}}^{{3}} {\text{z}}_{{{\text{ij}}}} {\text{n}}_{{{\text{ij}}}} $$

where z_ij_ is the depth of the ith sampling depth at the sampling time j, n_ij_ is the larval concentration at depth i and sampling time j, and N_j_ is the total number of larvae for a given species and developmental stage.

For each species, vertical distributions of larvae were compared between stages and between day and night using the statistical test developed by Beet et al. (2003)^89^, which allows to take into account the patchy distribution of zooplankton. The test statistic is:

$$ {\text{B}} = {\text{n}}\mathop \sum \limits_{{{\text{i}} = 1}}^{{\text{T}}} \mathop \sum \limits_{{{\text{j}} = 1}}^{{\text{D}}} \frac{{\left( {{\overline{\text{y}}}_{{{\text{ij}}}} - {\hat{\upmu }}_{{{\text{ij}}}} } \right)^{2} }}{{{\hat{\upmu }}_{{{\text{ij}}}} \left( {1 + {\hat{\text{c}}}{\hat{\upmu }}_{{{\text{ij}}}} } \right)}} $$

where T is the number of conditions (i.e. T = 2 for day-night comparison), D is the number of depths, ${\overline{\text{y}}}_{{{\text{ij}}}}$ is the average larval count of n replicates for condition i and depth j, ${\hat{\mu }}_{{{\text{ij}}}}$ and ${\hat{\text{c}}}$ are the maximum likelihood estimates of the mean (${\upmu }$_ij_) and dispersion coefficient (c_ij_) of a negative binomial distribution under the null hypothesis of a lack of difference in the vertical distribution profiles. The reference to the negative binomial distribution is recommended to describe the larval count data when the variance/mean ratio exceeds 1 due to aggregative nature of plankton^90^. The maximum likelihood estimates and the B statistics were obtained using the MATLAB script written by Beet et al. (2003)^89^.

To separate day and night hours, the use of PAR values was preferred to the official hours of sunrise and sunset. The nighttime was defined when PAR values in surface, mid-depth and bottom waters were close to zero, i.e. lower than 0.15 µmol m^−2^ s^−1^ (Fig. 1a, b). Thus in the bay of Douarnenez, the nighttime was from 23:00 to 06:00, and in the bay of Brest from 23:00 to 05:00. Hours of day-night transition (22:00 and 07:00 for Douarnenez, and 22:00 and 06:00 for Brest) were excluded from the analyses following Tapia et *al.* (2010)^88^. To compare the vertical distribution between developmental stages, daytime and nighttime were separated in order to minimize the effects of a potential diel migration. Because the statistical test applies only when the same number of replicates is available for each condition^89^, and to avoid any potential autocorrelation in larval vertical distribution between successive hours, statistical comparisons were carried out for groups of 5 daytime and 5 nighttime plankton profiles selected at random and without replacement from the complete datasets. This random draw procedure was repeated 10 times for each comparison, and ranges of values obtained for the test B and their associated probability (*p-values*) were retained, following the procedure used by Tapia et al. (2010)^88^. Differences were considered significant when 6 or more individual *p-values* (out of 10) were significant at the 0.05 significance level. The same procedure was used to compare the vertical distribution between developmental stages, except that the significance level for individual *p-values* was set as 0.01 to avoid any bias associated to multiple inferences due to pairwise comparisons between stages.

To assess the effects of larval vertical distribution on larval transport, instantaneous flux (F_zt_) at each depth and hour, as well as daily flux (F) of the larvae of the 3 species were calculated for the bay of Brest and the bay of Douarnenez in order to determine whether larvae enter or leave the bay according to Rowe and Epifanio (1994)^91^:

$$ {\text{F}}_{{{\text{zt}}}} = {\text{U}}_{{{\text{zt}}}} \times {\text{D}}_{{{\text{zt}}}} {\text{ and F}} = \frac{{\sum {\text{F}}_{{{\text{zt}}}} }}{{\text{n}}} \times 24 $$

where U_zt_ is the instantaneous longitudinal current velocity, D_zt_ is the instantaneous larval concentration at depth z and time t, and n the total number of hours sampled in the survey.

## Supplementary information

Supplementary information.
