# Supplementary material for: Vertical distribution of brittle star larvae in two contrasting coastal embayments: implications for larval transport
Source: Sci Rep. 2020 Jul 21;10:12033. doi: 10.1038/s41598-020-68750-4 (PMC7374168; doi:10.1038/s41598-020-68750-4)
Supplement: Supplementary file 1 — Supplementary information. [file 41598_2020_68750_MOESM1_ESM.pdf]

Supplementary information for Guillam et al., Vertical distribution of brittle star larvae in two contrasting coastal embayments: implications for larval transport

Morgane Guillam[1*], Claire Bessin[1], Aline Blanchet-Aurigny[2], Philippe Cugier[2], Amandine Nicolle[1,3], Éric Thiébaut[1], Thierry Comtet[1]

[1] Sorbonne Université, CNRS, Station Biologique de Roscoff, Laboratoire Adaptation et Diversité en Milieu Marin, UMR 7144 AD2M, CS90074, F-29688 Roscoff Cedex, France

[2] Ifremer, Centre de Bretagne, Département Dynamiques de l'Environnement Côtier (DYNECO), Laboratoire d'Ecologie Benthique (LEBCO), Technopole Brest Iroise, CS 10070, F-29280, Plouzané, France

[3] ENSTA Bretagne, Pôle STIC/OSM, 2 rue François Verny, F-29806 Brest Cedex 9, France

*Contact of corresponding author: morgane.guillam@sb-roscoff.fr

Supplementary methods: validation of the “local” high resolution model in the Bay of Brest.

The performance of the “local” high resolution MARS 3D model was assessed by comparing modelled vertical profiles of current velocities (Fig. 1a) with observed data measured by an Acoustic Doppler Current Profiler (ADCP; 600 khz WorkHorse Sentinel) (Fig. 1b).
The visual comparison between the observed and simulated vertical profiles of current velocities showed a good adequacy between the two time series in terms of maximum current velocities and representation of the tidal cycle (Fig. 1). Both series showed the lack of vertical structure in current velocities except in the bottom boundary layer. A highly significant correlation was observed between simulated and observed current velocities (Pearson correlation coefficient $r = 0.85$; $n = 4850$; $p < 0.001$) (Fig. 2a).
A Taylor diagram (Fig. 2b) was plotted to quantify concisely the degree of correspondence between the simulated and observed data by means of three statistics: the Pearson correlation coefficient, the root mean square error (RMSE), and the standard deviation (SD)[1]. A high degree of correspondence occurs when (1) the Pearson correlation coefficient r is high; (2) the RMSE in the simulated data is low; and (3) the standard deviations (SD) of simulated and observed data are similar, indicating a similar pattern of temporal variations. The closer the observed and simulated points are, the better the match between the simulated and observed data. In the present case, the highly significant correlation coefficient ($r = 0.85$; $n = 4850$; $p < 0.001$), the low RMSE value (0.12 $m.s^{-1}$) and the very close SD values for the simulated and observed data (0.21-0.22 $m.s^{-1}$ *vs*. 0.22-0.23 $m.s^{-1}$, respectively) showed that the “local” high-resolution hydrodynamic model reproduced well the observed instantaneous currents in the Bay of Brest and can therefore be used in our study with confidence to calculate larval fluxes.

Reference.

1. Taylor, K. E. Summarizing multiple aspects of model performance in a single diagram. *J. Geophys. Res.* **106**, 7183–7192 (2001).

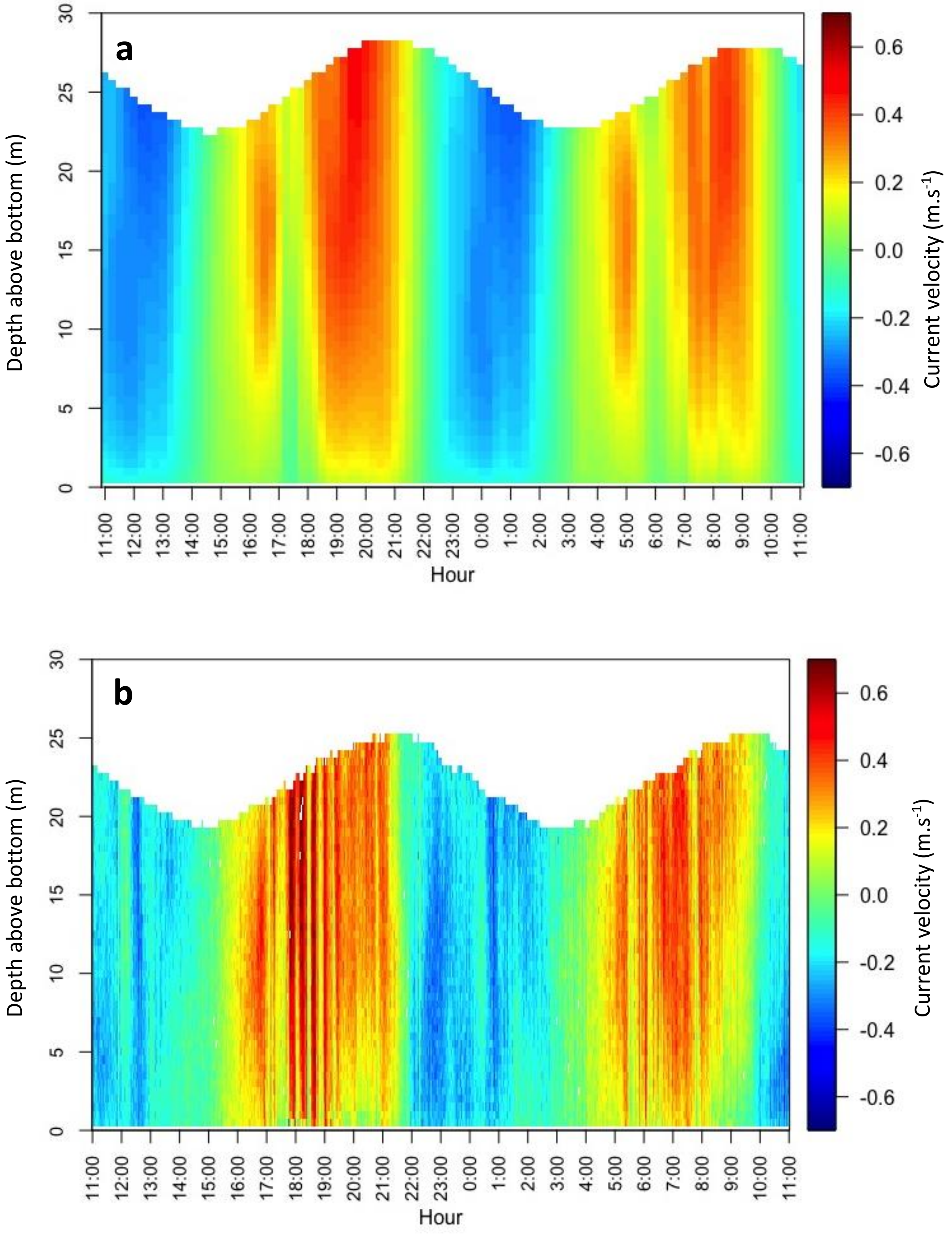

Figure 1. Current velocities from the "local" 170-m resolution model (a) and the Acoustic Doppler Current Profiler (ADCP) (b); positive values indicate eastward currents.

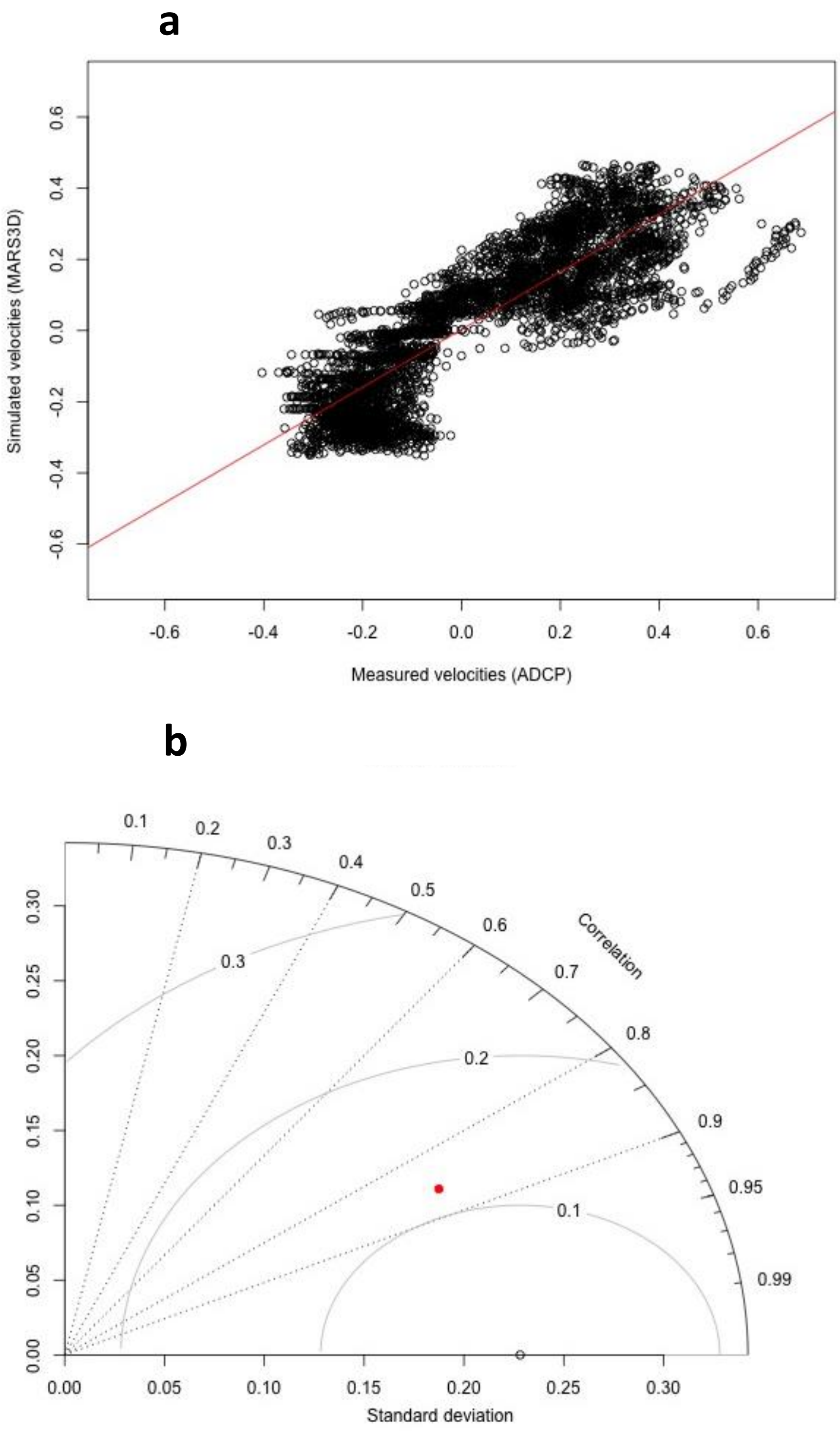

Figure 2. Comparison between simulated and observed current velocities in the bay of Brest. (a) Relationship between simulated currents and ADCP measurements; (b) Taylor Diagram. (a) The red line corresponds to the regression line. (b) The X and Y axes correspond to the standard deviation that should be read as the radial distance from the origin (contours not drawn). The dashed lines correspond to the azimuthal angles related to the values of Pearson correlation coefficient. The solid contours correspond to the isolines of the RMSE. The white point corresponds to the observed data and the red point corresponds to the simulated data.

Supplementary Figures.

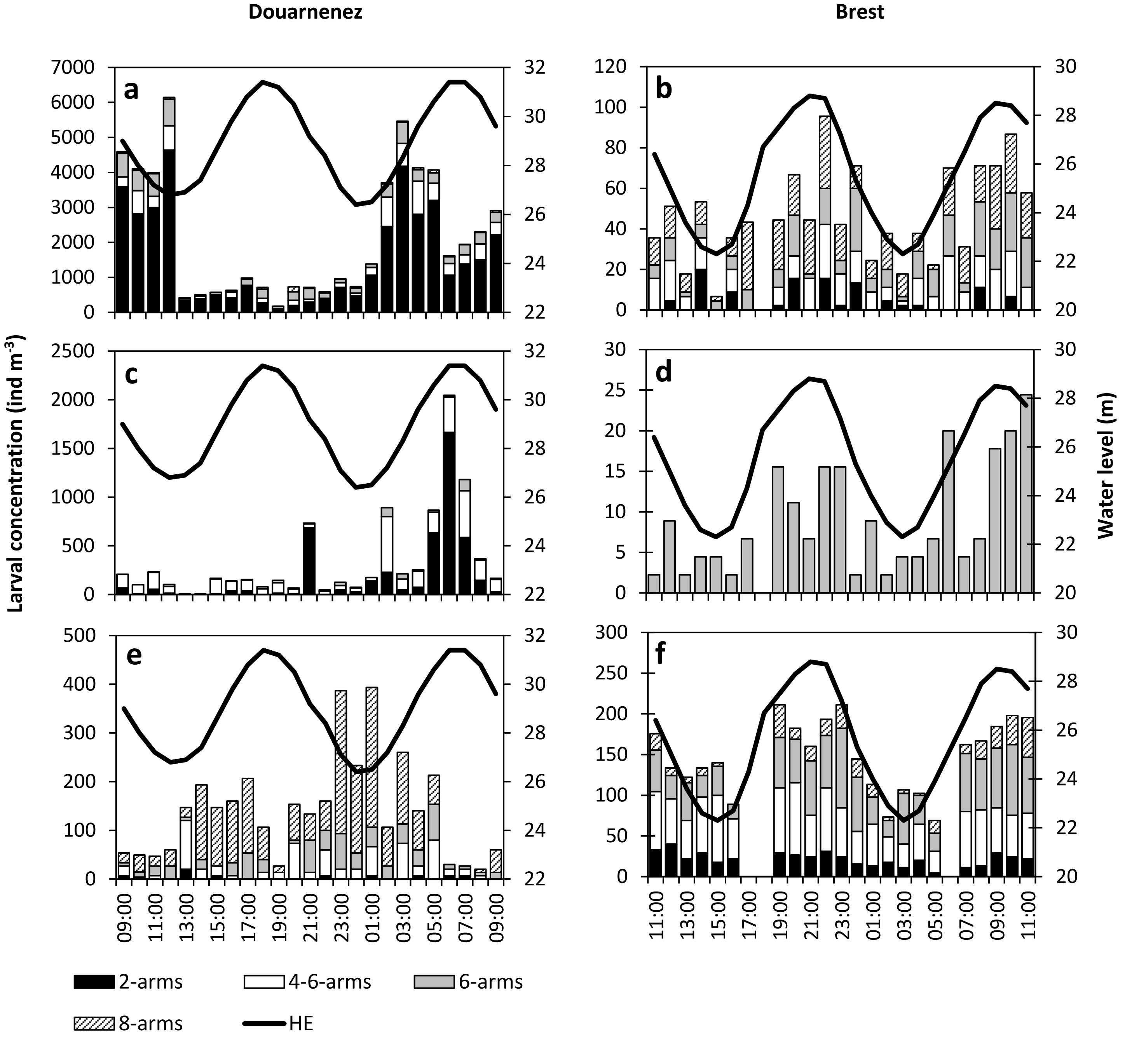

Supplementary Figure S1. Depth-cumulated larval concentration of (a, b) *Ophiocomina nigra,* (c, d) *Amphiura filiformis,* and (e, f) *Ophiothrix fragilis* in the bay of Douarnenez (left column) and the bay of Brest (right column) in relation to tidal-cycles. The black line represents the water level (m). The proportion of the different developmental stages is indicated (see the Methods section for the description of these stages).

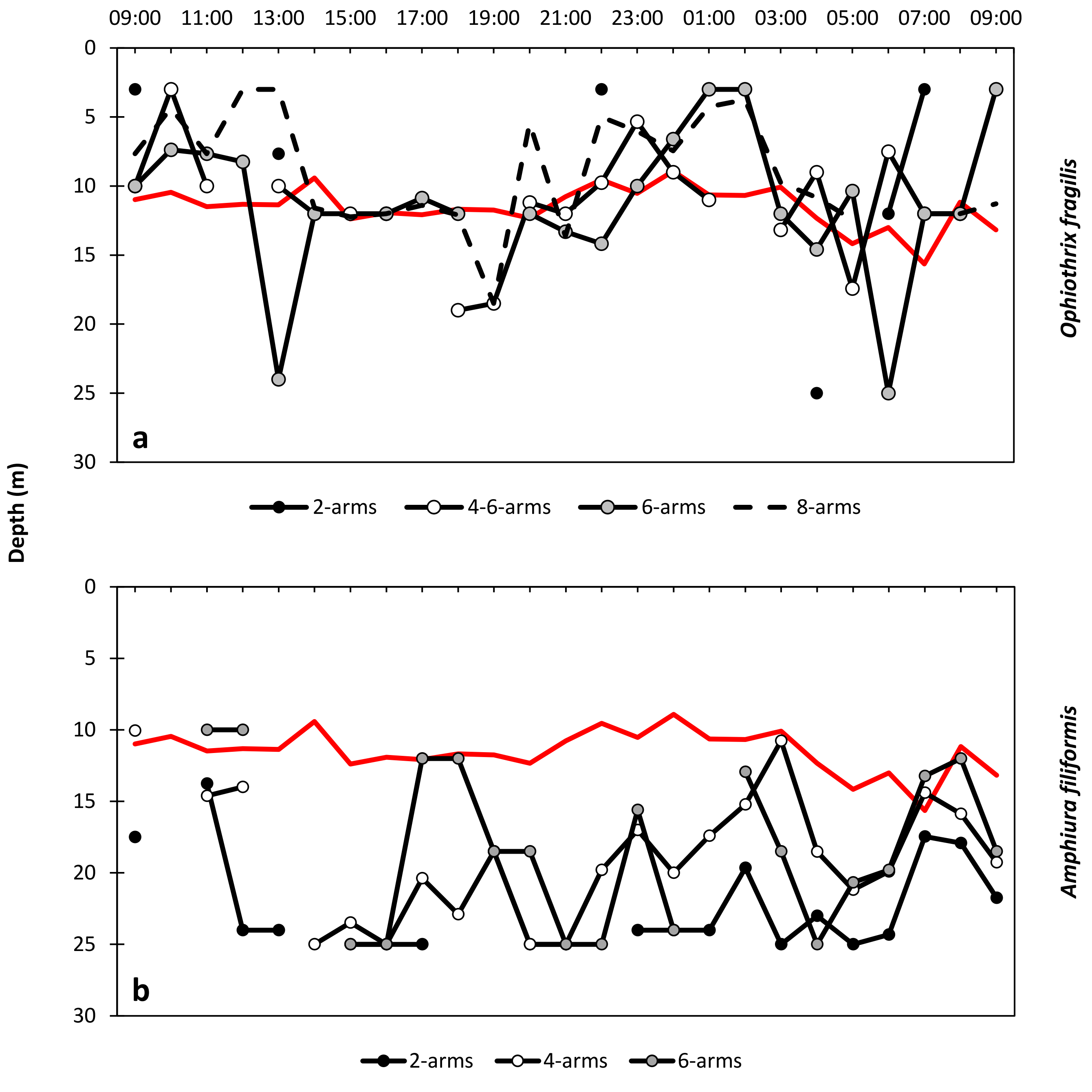

Supplementary Figure S2. Mean Depth Distribution (MDD) of the different larval stages of (a) *Ophiothrix fragilis* and (b) *Amphiura filiformis* in the bay of Douarnenez. Red line indicates the thermocline.
